# Application of Bioactive Quercetin in Oncotherapy: From Nutrition to Nanomedicine

**DOI:** 10.3390/molecules21010108

**Published:** 2016-01-19

**Authors:** Ju-Suk Nam, Ashish Ranjan Sharma, Lich Thi Nguyen, Chiranjib Chakraborty, Garima Sharma, Sang-Soo Lee

**Affiliations:** 1 Institute for Skeletal Aging & Orthopedic Surgery, Hallym University-Chuncheon Sacred Heart Hospital, Chuncheon 200704, Korea; jsnam88@hallym.ac.kr (J.-S.N.); boneresearch@hallym.ac.kr (A.R.S.); lichbio@gmail.com (L.T.N.); 2 Department of Bio-informatics, School of Computer and Information Sciences, Galgotias University, Greater Noida 203201, India; drchiranjib@yahoo.com; 3 Amity Institute of Nanotechnology, Amity University Uttar Pradesh, Noida, Uttar Pradesh 201313, India

**Keywords:** quercetin, anticancer, oncotherapy, bioavailability, nanoformulation, phytochemical

## Abstract

Phytochemicals as dietary constituents are being explored for their cancer preventive properties. Quercetin is a major constituent of various dietary products and recently its anti-cancer potential has been extensively explored, revealing its anti-proliferative effect on different cancer cell lines, both *in vitro* and *in vivo*. Quercetin is known to have modulatory effects on cell apoptosis, migration and growth via various signaling pathways. Though, quercetin possesses great medicinal value, its applications as a therapeutic drug are limited. Problems like low oral bioavailability and poor aqueous solubility make quercetin an unreliable candidate for therapeutic purposes. Additionally, the rapid gastrointestinal digestion of quercetin is also a major barrier for its clinical translation. Hence, to overcome these disadvantages quercetin-based nanoformulations are being considered in recent times. Nanoformulations of quercetin have shown promising results in its uptake by the epithelial system as well as enhanced delivery to the target site. Herein we have tried to summarize various methods utilized for nanofabrication of quercetin formulations and for stable and sustained delivery of quercetin. We have also highlighted the various desirable measures for its use as a promising onco-therapeutic agent.

## 1. Introduction

The medicinal properties of various phytochemicals, such as anti-oxidative, anti-inflammatory, antimicrobial and anticancer activities, have been known to mankind since ancient times. Flavonoids are well known phytochemicals possessing medicinal properties and are found in fruits and vegetables as secondary metabolites. To date, around 9000 types of flavonoids are classified in natural foods [1,2]. Quercetin is among these dietary flavonoids and is attracting increasing interest as a novel medicinal biomolecule with diverse therapeutic properties [3]. In a general daily diet comprising fruits and vegetables, quercetin is available as conjugated isoforms bound to alcohols and sugars [4]. These isoforms get hydrolyzed in the gastrointestinal tract and are absorbed and metabolized into quercetin aglycone and its other derivatives [5]. Among identified flavonoids quercetin is well known for possessing potent antioxidant activity, due to its ability to eliminate highly reactive oxygen species (O_2_^−^and ONOO^−^) [6,7]. Henceforth, the augmentation of mutated cell apoptosis by modulating cell signaling pathways is reported, which may result in the inhibition of cancer growth [8,9].

**Figure 1:**
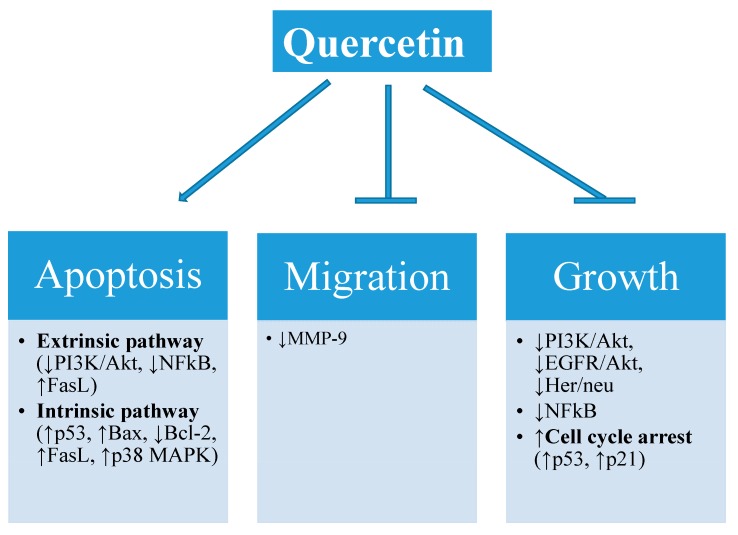
Cellular processes modulated by quercetin (↓: downregulate and ↑: upregulate).

Worldwide, cancer has emerged as a foremost challenge for clinicians and is causing high mortality rates, both in developed as well as developing countries. Globally, in 2015, around 1.6 million new cases of cancer are expected to be diagnosed [10]. Available statistics account for more than 100 types of cancer known so far, including breast, colorectal, prostate, liver and lung cancers [11]. Regardless of new therapeutic approaches being progressively developed, effective cancer treatment therapies are still needed. Recently, it has been observed that a diet rich in fruits and vegetables reduces the risk of various types of cancer [12,13]. Thus, in the search for novel therapeutic drugs various studies have tried to explore the anticancer potential of natural compounds available from fruits and vegetables, such as phytochemicals [14,15,16]. As documented, these phytochemicals showed anticancer activities by modulating various cellular processes and interfering with cancer progression and metastasis (Figure 1) [17,18]. Among phytochemicals, quercetin has been reported to possess apoptotic activities against different cancer lines such as leukemia HL-60 [19], SW-480 colon cancer [20], 4T1 murine mammary cancer [21] and A431 epidermoid tumor cells [22]. However, the application of quercetin in pharmaceutics is still limited due to its poor bioavailability, hydrophobic nature and low stability [23]. Furthermore, quercetin tends to undergo high levels of enzymatic degradation in the gastrointestinal tract and have a low circulation time in the body [24,25]. Some studies also reported the toxic effects of quercetin at high doses in clinical phase trials [26]. Therefore, development of modified dosage forms of quercetin with increased bioavailability, long term stability, prolonged circulation time and decreased toxic effects at low doses is advocated.

Nanotechnology-based drug delivery systems, loaded with phytochemicals, have recently gained interest in pharmaceutics due to their recuperative bioavailability and absorption potential [27,28]. Moreover, nanodelivery systems also protect the therapeutic molecule from being enzymatically metabolized, henceforth, increasing its stability and circulation time [29,30]. Conjugation of cancer cell specific targeting moieties with these nanodrugs also enable efficient selective uptake of drug by cancer cells as compared to normal cells [31]. Keeping all this in view, herein, we discuss the promising role of nanotechnology-based quercetin delivery systems for cancer therapy and the various merits and demerits associated with these approaches.

## 2. Chemistry of Quercetin and Its Derivatives

**Figure 2:**
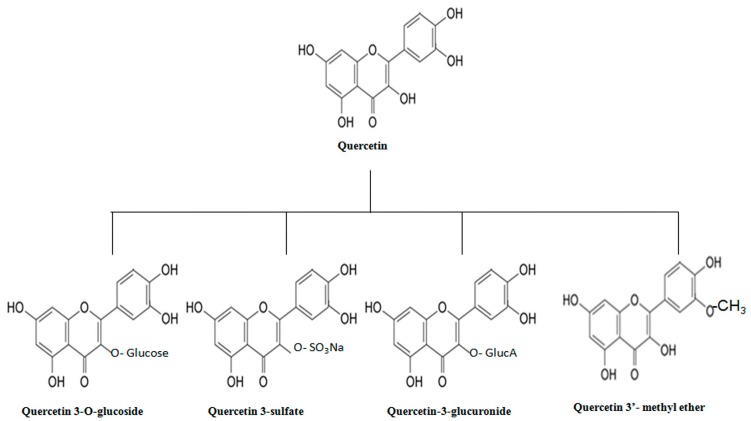
Structure of quercetin and its derivatives.

Quercetin (3,3′,4′,5,7-pentahydroxyflavone, Figure 2), is a naturally occurring flavonoid (flavone means yellow colour) and is a derivative of a flavone (2-phenylchromen-4-one). It contains five hydroxyl groups that are responsible for its biological activities and derivative diversification. Flavonoids generally consist of two benzene rings linked by pyran or pyrone rings [32]. In addition, the conformational analysis of quercetin showed the presence of 12 conformations of this molecule having Gibbs energies in the range of 0 to 5.33 kcal/mole. Furthermore, quercetin displays strong intramolecular H-bonding, explaining its biological multi-functionality and renders it the ability to form strong complex interactions, even with metals, affecting its bioavailability and transport [33]. Among these H-bonds, two bonds are with carbonyl groups and third one is between hydroxyl groups [34]. Naturally occurring quercetin acts as an auxin transport inhibitor and prevents the bilateral growth of plant embryos [35]. The most widely present derivatives of quercetin are glycosides and ethers [36,37]. Quercetin *O*-glycosides contains *O*-glycosidic bonds at the C-3 carbon hydroxyl group. Furthermore, a rare quercetin derivative form, a C-glycoside whose glycosylation site is the C-6 carbon, was also found in *Ageratina calophylla* [38]. Another rare derivative of quercetin, identified in the red alga *Acanthophora spicifera* and grapes (*Vitis vinefera*), is quercetin3-*O*-α-l-fucopyranoside where quercetin is attached to α-l-fucopyranosyl moiety through a glycosidic linkage at the C-3 position [39,40]. Moreover, the derivatives of quercetin glycosides may also contain acyl and sulfur substituents in addition to sugar moieties. In the case of ether derivatives the hydroxyl groups of quercetin are attached with alcohols via ether bonds. Although quercetin is lipophilic, the glycosylation of quercetin derivatives can increase the hydrophilicity and enable the molecules to transport through all parts of the plant [41].

Previous studies identified the antioxidant abilities of quercetin, attributed by the presence of hydroxyl group in the A ring and a catechol group in the B ring [42]. This property enables quercetin to eliminate free oxygen species present in the body by transferring hydrogen or electrons or by chelating metal ions, thereby inhibiting enzymatic activities. Such scavenging of reactive oxygen species reduces inflammation and protects the cells with oxidative stress caused due to smoking or excessive exercise. Quercetin is also well known inhibitor of lipid peroxidation, preventing oxidation of low density lipoproteins (LDL) and damage of lipid membrane. Human cell membranes and LDL possess α-tocopherol (primary oxidant) which provides protection from deleterious effects of oxidation. Flavonoids can therefore slow the oxidation of lipoproteins by contributing the hydrogen atom to this α-tocopheryl radical [43,44]. In addition, it also elevates the level of glutathione and prevents the formation of free radicals. In consideration of the above facts, the antioxidant and free radical scavenging properties of quercetin have been studies to a vast extent, displaying the potential therapeutic benefits of quercetin.

## 3. Sources, Absorption and Metabolism of Quercetin

Quercetin is mainly found as glycosides in the edible parts of plants (such as quercetin glucosides or quercetin rutinoside, Figure 2). It is found in a variety of plants including berries, tea leaves, onion, broccoli and other fruits and leafy vegetable, but is mostly extracted from *Sophoro japonica* L*.* [45,46,47]. These fruits and vegetables are among the main food constituents in Western diet containing flavonoids (~350 ppm, expressed as aglycones) [48]. Besides, red wine, black tea and other fruit juices are also considered rich in dietary quercetin [49]. The average daily consumption of flavonoids, including flavones, flavanones, flavonols, catechins, anthocyanins, and biflavans in the United States amounts to around 1 g/day (expressed as quercetin equivalents), among which 160–175 mg/day is accounted for only by flavanones, flavones, and flavonols based on seasonal variations [50]. Individuals consuming quercetin-rich fruits and vegetables, such as tomatoes, may obtain about 200–500 mg/day of quercetin as a dietary constituent [51]. On the other hand, average intakes as low as <5 mg to 40 mg/day are also reported in some countries like Japan and Australia [52,53].

The therapeutic efficiency of quercetin may be defined by its bioavailability that is the amount of pharmacologically active drug absorbed upon oral administration [54]. Following ingestion of quercetin, the intestinal bacteria exert glycosidase activity and hydrolyze the sugar unit from β-glycosidase derivatives of quercetin to release quercetin in its aglycone form [55]. Quercetin aglycone is further absorbed via the stomach [56] or small intestine [57], either by passive diffusion or organic anion transporting polypeptide (OATP)-mediated absorption [58]. Absorbed quercetin aglycone is then metabolized into other pharmacologically active *O*-methyl, glucuronide or sulfate derivative forms in human plasma, such as 3′-methyl ether, quercetin 3-*O*-β-d-glucuronide (Q3GA) and quercetin-3′-sulfate (Figure 2) [5,59]. Intake of quercetin rich foods, such as onions, may increase the concentration of quercetin derivatives in plasma up to micromolar levels which are then metabolized in the liver and other organs [60]. Walle *et al.*, suggested that a majority of quercetin metabolites in plasma are further metabolized in the lungs and are eliminated as CO_2_ (23.0%–81.1% of the oral quercetin dose). They also reported that only 3.3%–5.7% and 1.6%–4.6% of quercetin is recovered in urine and feces, respectively [61]. It was also observed that upon single gastric treatment of free quercetin at a dose of 50 mg/Kg body weight in male Sprague-Dawley rats, only 0.27 μg/mL free quercetin was quantified in plasma while 93% of the quercetin was metabolized within a time span of 1 h [62]. Studies also revealed the conversion of glucuronide and sulfate derivatives of quercetin into phenolic acids by colonic microflora before excretion [63]. In a study, about 21 different types of quercetin metabolites were recovered from urine after onion intake [64]. A previous study has also reported the presence of less than 8% of intragastric treated quercetin in kidneys and liver of rats [4]. The quercetin metabolism process within the body is thus quite rapid and consequently the available circulation time of the active quercetin molecule and its bioavailability is limited. Due to this rapid metabolic process, the observed pharmacological effect of quercetin *in vitro* is dissimilar to a great extent with that *in vivo*. Also, quercetin has low solubility in water (about 1 µg/mL) which further affects its therapeutic potential. Additionally, the solubility of quercetin in gastric and intestinal fluid was reported to be 5.5 µg/mL and 28.9 µg/mL, respectively. Henceforth, there is a need to increase the aqueous solubility and delay the metabolism of quercetin to maintain its levels in blood and other tissues for a prolonged time.

Besides medicinal properties, quercetin has also been investigated for its potential toxic effects such as pro-oxidant activity, mutagenicity, genotoxicity, inhibition of enzymes responsible for hormone metabolism, and mitochondrial toxicity [65]. Crebelli *et al.*, demonstrated that plasma samples collected from rats, following oral administration of quercetin, lack mutagenic properties whereas fecal and urine samples displayed mutagenicity, suggesting that upon absorption quercetin may get metabolized into non-mutagenic derivatives [66]. Some studies also reported that the quercetin reveals renal toxicity at high doses during a phase I clinical trial [26]. On the contrary, Harwood *et al.* reviewed the non-toxic nature of quercetin *in vivo* compared to its *in vitro* behavior [67]*.* These uncertainties create a need for more advanced toxicity and safety analysis for quercetin before its application as therapeutic molecule in clinics.

## 4. Effect of Quercetin on Cancer Cell Biology

### 4.1. Inhibition of Cell Growth

Quercetin is known to exert antiproliferative effects on various types of cancer, both *in vitro* and *in vivo*. For example, *in vitro* studies demonstrated growth inhibitory effects of quercetin in the leukemia cell lines L1210 and P-388 [68], breast cancer cells [21], colon cancer cells COLO 20DM [69], ovarian cancer cells OVCA 433 [70], liver cancer cells HepG2 [71], epidermoidal cancer cells A431 [22] and gastric cancer cells [72]. Moreover, the antineoplastic activity of quercetin towards Walker carcinoma 256 was documented by Edwards *et al.* [73]. An antiproliferative effect of orally administered quercetin on pancreatic cancer cells was also observed *in vivo* by Angst [74]. The possible interaction of quercetin with signaling pathways which are responsible for cancer growth may lead to growth inhibition. Some of the signaling pathways explored are P13K/Akt, Her-2/neu, Wnt/β-catenin and EGFR. This flavonoid was found to inhibit mammalian target of rapamycin (mTOR) activity which gets hyperactivated during cancer and controls essential cell growth pathways, autophagy and biosynthesis of proteins, and interfere with the activation ofPI3K/Akt signaling pathway [75]. Suppression of P13K/Akt pathway was also demonstrated in other cell lines like breast cancer cell HCC1937 [76], SkBr3 cells [77], liver cancer cell HepG2 [71], and HL-60 leukemia [19], upon quercetin treatment. In addition, quercetin was also able to suppress the tumor growth in Dalton’s lymphoma mice by downregulating P13K-Akt-p53 pathway along with glycolytic metabolism [78]. Besides suppressing P13K/Akt pathway, quercetin can also downregulate human epidermal growth factor receptor 2 (Her-2/neu) in HER-2/neu overexpessing breast cancer cells which resulted in growth inhibition [77]. EGFR signaling pathway was also observed to be inhibited by quercetin in Sprague-Dawley male rats [79]. Moreover, quercetin induced inhibition of Wnt/β-catenin signaling pathway through the induction of Wnt antagonist Dickkopf (DKK) has also been illustrated which decreased the cell viability of 4T1 murine mammary cancer cells [21]. The involvement of quercetin regulated Wnt/beta-catenin signaling pathway in exhibiting antitumor effect in SW-480 colon cancer cells is also documented [20].

Besides modulating signaling pathways for proliferation, quercetin is also known to interfere with normal cell cycle progression which can lead to growth inhibition. Mu *et al.*, evaluated that quercetin arrest cell cycle progression at G1 phase in HepG2 cells. Furthermore, they also showed that the growth inhibitory effect of quercetin is due to the increased expression of p21 and p27 (Cdk inhibitors) and p53 (tumor suppressor) [80]. Other studies also reported the cell cycle arrest at G2/M in HeLa cells [81], A549 lung cancer cells [82], SK0V ovarian cancer cells, U2OS osteosarcoma cells [83], HSC-3 and TW206 oral cancer cells after quercetin treatment [84]. From the mentioned literature one may conclude that quercetin can act as a potential growth inhibitor of tumor cells by regulating various cellular growth-associated biochemical events and arresting the cells during cell cycle events.

### 4.2. Inhibition of Metastasis

The utmost danger of cancer is its spread from one organ or part to other within a body through metastasis. Metastasis is related with the production of matrix metalloproteinases (MMPs) enzymes which are responsible for degrading extracellular matrix proteins in a variety of cells or tissues they encounter. They are classified into four types: stromelysins, gelatinases, collagenases and membrane type MMPs [85]. Earlier studies reported that MMPs are required for the invasion/metastasis of cancer cells, such as MMP-9 and MMP-2. Therefore, suppression of MMPs in cancer cells can diminish the chances of metastasis and can be a useful tool in cancer treatment. The potential of quercetin in inhibiting MMPs secretion was reported in various studies. Lin *et al.*, identified that quercetin can prevent metastasis of breast cancer cells by suppressing the activation and migration of MMP-9 in 12-*O*-tetradecanoyl phorbol-13-acetate (TPA)-treated MCF-7 cells [86]. Quercetin also inhibited upregulation of MMP-9 by tumor necrosis factor (TNF)-α in JB6 P+ mouse epidermal cells [87]. Along with MMP-9, quercetin also decreased the secretion of MMP-2 in A431 epidermoid cancer cells [22], MiaPaCa-2 pancreatic cancer cells [88].

### 4.3. Induction of Apoptosis

Apoptosis is programmed cell death which plays a critical role in various physiological processes [89]. Specific gene activities regulate complex signaling pathways known to be responsible for cell apoptosis. Dysregulation of the apoptotic signaling results in apoptotic defects which can lead to cancer progression [90]. For cancer treatment, the major problem is the resistance of cancer cells against chemotherapeutic agents. Therefore, for efficient cancer treatment it is necessary to comprehend regulation of apoptotic signaling pathways via chemotherapeutic agents such as quercetin. There may be two pathways responsible for apoptosis: intrinsic (mitochondria mediated) or extrinsic (mediated by signals from other cells) (Figure 1). Studies have revealed that quercetin induces apoptosis in various types of cancer cells by regulating both extrinsic and intrinsic factors. Chien *et al.*, demonstrated that quercetin tends to increase the synthesis of Bax (pro-apoptotic protein) and decrease the synthesis of Bcl-2 (anti-apoptotic protein) in MDA-MB-231 breast cancer cells [91]. Induction of apoptosis through AMP-activated protein kinase (AMPK) along with p-53 dependent apoptotic cell death was observed in HT-29 colon cancer by quercetin [92]. Seo *et al.*, also showed quercetin induces p-53 dependent apoptotic cell death in ERalpha-negative breast cancer cells [93]. Conversely, quercetin can induce p-53 independent apoptotic pathway in HT-29 colon cancer cells via AMPK/p38 signaling pathway [94]. Although various studies have shown the apoptotic activity of quercetin, there is further need to understand more detail about the mode of action and mechanisms responsible for quercetin induced apoptosis. Some of the quercetin induced anticancer effects in different cell lines are summarized in Table 1.

**Table 1:** Effects of quercetin on cancer types and cell signaling involved.

Organ/Tissue	Carcinogen/Cancer Cell Lne	Mode of Study/Model System	Effect/Signaling Mechanism	Ref.
Breast	MCF-7 breast cancer cells	*In vitro*	Induce antiproliferative effect and apoptosis by increased Bcl-2 and decreased Bax expression	[95]
	HCC1937 breast cancer cells	*In vitro*	Induce antiproliferative effect via PI3K-Akt/PKB pathway	[76]
	SK-Br3 breast cancer cells	*In vitro*	Growth inhibition by decreasing level of Her-2/neu protein and inhibition of PI3K-Akt signaling pathway	[77]
	SK-Br3 breast cancer cells	*In vitro*	Induce antiproliferative effect by suppressing hypoxia-inducible factor-1alpha (HIF-1alpha) accumulation and reduced vascular endothelial growth factor (VEGF) secretion	[96]
	4T1 breast cancer cells	*In vitro*	Induce antiproliferative effect and apoptosis by regulating Wnt/β-catenin signaling pathway	[21]
	TPA-treated MCF-7 breast cancer cells	*In vitro*	Prevents metastasis by inhibiting TPA-induced PKC δ/ERK/AP-1-dependent matrix metalloproteinase-9 activation and migration	[86]
	MDA-MB-231 breast cancer cells	*In vitro*	Growth inhibition by arresting cell cycle and inducing and inducing apoptosis by regulating mitochondrial- and caspase-3-dependent pathways	[91]
	MCF-7 and MDA-MB-231 breast cancer cells	*In vitro*	induces apoptosis through suppression of Twist via p38MAPK pathway	[97]
	ERalpha-negative breast cancer cells	*In vitro*	Induce antiproliferative effect and apoptosis via p53-dependent pathway	[93]
Pancreas	MIA PaCa-2 and BxPC-3 pancreatic cancer cells	*In vitro* and *in vivo* (nude mouse model)	Induce antiproliferative effect and apoptosis. Inhibits tumor growth	[74]
Colon	CX-1 colon cancer cells	*In vitro*	Induce antiproliferative effect by suppressing hypoxia-inducible factor-1α (HIF-1α) accumulation and reduced vascular endothelial growth factor (VEGF) secretion	[96]
	SW480 colon cancer cells	*In vitro*	Growth inhibition via inhibiting cyclin D(1) and survivin expression as well regulating Wnt/β-catenin signaling pathway	[20]
	HT-29 and HCT116 colon cancer cells	*In vitro*	regulates the sestrin 2-AMPK-p38 MAPK signaling pathway and inducing apoptosis via increasing the generation of intracellular ROS in a p53-independent manner	[94]
	HT-29 colon cancer cells	*In vitro* and *in vivo* (male nude mice)	Induces apoptosis via AMPK signaling pathway and reduce tumor volume	[92]
Prostate	LNCaP prostate cancer cells	*In vitro*	Induce antiproliferative effect by suppressing hypoxia-inducible factor-1α (HIF-1α) accumulation and reduced vascular endothelial growth factor (VEGF) secretion	[96]
	Chemically induced prostate cancer	*In vivo* (Sprague-Dawley male rats)	Suppress tumor progression by inhibiting the EGFR signaling pathway, regulating cell adhesion molecules and decreased snail, slug, and twist mRNA levels	[79]
	PC-3 prostate cancer cells	*In vitro*	Prevent metastasis via regulating EGFR/PI3k/Akt/ERK1/2 pathway and by suppressing transcriptional repressors Snail, Slug and Twist	[98]
Liver	HepG2 hepatic cancer cells	*In vitro*	Induce antiproliferative effect by downregulating phosphatidylinositol 3-kinase (PI3K) and protein kinase C (PKC) via induction of p53	[71]
	HepG2 hepatic cancer cells	*In vitro*	Induce growth inhibition by cell cycle arrest at G1 phase and increasing levels of Cdk inhibitors p21 and p27 and tumor suppressor p53	[80]
Lymphatic system	Dalton’s lymphoma ascite cell line	*In vivo*	Inhibiting cancer growth by down-regulation of PI3K-Akt1-p53 pathway and glycolytic metabolism	[78]
	Dalton’s lymphoma ascite cell line	*In vivo*	Induction of apoptosis and modulation of PKC signaling with the reduction of oxidative stress	[99]
Salivary glands	ACC salivary cancer cells	*In vitro*	Induce antiproliferative effect and apoptosis by down-regulating the PI3K/Akt/IKK-alpha/NF-kappaB signaling pathway.	[100]
Ovary	SKOV3 oarian cancer cells	*In vitro*	Inhibiting cell growth by decreasing cyclin D1 expression level linked to alterations in G1/S phase	[83]
	SKOV3 oariancancer cells	*In vitro* and *in vivo* (SKOV-3 xenograft mice model)	Inducing apoptotic effect of tumor necrosis factor-related apoptosis-inducing ligand (TRAIL) via ROS mediated CCAAT enhancer-binding protein homologous protein (CHOP)-death receptor 5 pathway	[101]
Bone	U2OS osteocarcoma cells	*In vitro*	Inhibiting cell growth by decreasing cyclin D1 expression level linked to alterations in G1/S phase	[83]
Cervix	HeLa cervical cancer cells	*In vitro*	Induce cell growth inhibition and mitochondria mediated apoptosis via p53 induction and NF-kappaB inhibition	[81]
	HeLa cervical cancer cells	*In vitro*	Induce antiproliferative effect and apoptosis by promoting cytochrome release, ROS accumulation and inhibiting anti-apoptotic AKT and Bcl-2 expression. Also perform cell arrest at G2/M phase	[102]
Lung	A549 lung cancer cells	*In vitro*	quercetin-3-glucuronide and quercetin-3'-sulfate enriched plasma induces cell growth inhibition by cell cycle arrest at the G (2)/M phase via downregulating cdk1 and cyclin B expression	[82]
Skin	JB6 P+ mouse epidermal cancer cells	*In vitro*	inhibit TNF-alpha-induced upregulation of MMP-9 and cell migration via p13K/Akt signaling pathway	[87]
Brain	U87 and U251 glioma cells	*In vitro/in vivo* (C6 glioma xenograft models)	Induce anti proliferative effect and autophagy	[103]
	U373MG glioblastoma cells	*In vitro*	Induce cell growth inhibition through mitochondria mediated apoptosis via activating Caspase-, caspase-7, JNK and p53 level. showed cell cycle arrest at sub-G1 phase	[104]

## 5. Enhancing Bioavailability and Bioactivity of Quercetin Using Nanoparticles and Its Application in Cancer Treatment and Diagnosis

**Figure 3:**
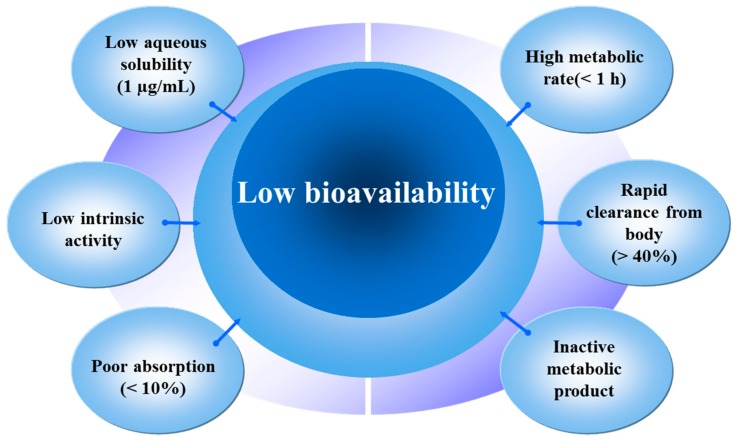
Limitations of quercetin as an anti-cancer drug.

Although, quercetin has been studied as a potential candidate for cancer treatment it has certain limitations. Some of these limitations are illustrated in Figure 3. Hence, to overcome the restrictions offered by phytochemicals, one of the widely explored method is to deliver them through polymers, liposomes, chitosan and other drug carriers. These delivery carriers are used for enhancing their solubility in water, absorption in body, circulation time and target specificity. Some of the biodegradable materials commonly used for this purpose are discussed below.

### 5.1. Silica Nanoparticles

The use of mesoporous silica nanoparticles as a promising drug delivery system has been reported in various studies due to its low *in vivo* toxicity [105], high stability and ability to be functionalized by various ligands to achieve targeted drug delivery [106,107,108]. In addition, mesoporous silica nanoparticles have high surface area and ordered tunable porosity rendering them high drug loading efficiency with better release kinetics [109,110]. The reported bio-distribution and excretion profile of mesoporous silica nanoparticles makes them an opportunistic nanomaterial for controlled drug delivery [111,112]. Zhang *et al.*, showed increased oral bioavailability, ~154% compared to free poorly water soluble drug, by loading them onto mesoporous silica nanoparticles [113]. Physical adsorption and solvent evaporation are the most employed silica nanoparticles based drug loading methods [114,115]. Furthermore, Catauro *et al.* reported the sol-gel synthesis method of silica-quercetin hybrid material to counteract peri-implant diseases, in which the C-ring of quercetin was modified structurally while the *O*-catechol ring was intact to perform antioxidant activity [116]. As discussed earlier that the antioxidant property of quercetin provides it a better therapeutic potential against cancer, similar kind of silica-quercetin hybrid which retains their antioxidant property can be suggested as a potent anticancer agent. Additionally, aminopropyl functionalized mesoporous silica nanoparticles loaded with quercetin were also synthesized for topical application as a chemopreventive agent [117].

Based on the structure, another type of silica nanoparticles are silicon dioxide (SiO_2_) nanoparticles having smaller surface area compared to mesoporous silica nanoparticles. They are mainly employed to immobilize the therapeutic molecules to modulate their controlled release and thus enhancing their biological activities [118]. In 2011, US Food and Drug Administration (FDA) approved silica nanoparticles for first human clinical trial aimed at targeted imaging of cancer, reflecting the clinical acceptance of silica nanoparticles in near future [119,120]. Although the advances in silica based quercetin delivery system are rousing there are overwhelming tasks for its clinical application which still need to be solved. For example, there is a need for monitored bioimaging to understand the function of delivery system more precisely. This may assist in improving their efficiency of more controlled quercetin release in biological systems. Furthermore, toxicity investigation of silica-based drug delivery systems for quercetin is also a prerequisite. Thus, a more refined and successful strategy for silica-based quercetin delivery system may be expected in future, utilizing the benefits offered by the silica nanoparticles against cancer.

### 5.2. PLGA and PLA Nanoparticles

Poly lactic-co-glycolic acid (PLGA) and poly(d,l-lactic acid) (PLA) are FDA and European Medicine Agency (EMA) approved biodegradable polymers which first get hydrolyzed into lactic and glycolic acids and finally metabolized to CO_2_ and H_2_O via the Krebs cycle [121]. Attributed to their biocompatibility, these polymers have been immensely used for the preparation of drug loaded nanoparticles for the treatment of various diseases. The physicochemical properties of these polymers have been amended by synthesizing their various derivatives and co-polymers. Synthesized derivatives have varying molecular weights with diverse properties like modulated size, drug loading ability, controlled drug release, nanoparticle uptake efficiency, bio-distribution and circulating half-life of the nanodrug composites [122,123,124]. In numerous studies, anti-cancer agents have been encapsulated within PLGA nanoparticles and have shown significant improvement in drug uptake and cancer growth suppression. For example, significant enhancement in A549 cell (a human lung adenocarcinoma epithelial cell line) cytotoxicity was observed by quercetin encapsulated PLGA nanoparticles in combination with etopside-loaded PLGA nanoparticles, as compared to free drugs [125]. Similarly, approximately 50% (in 2 days) and 40% (in 5 days) reduction of breast cancer cells was reported upon treatment with free quercetin and PLA-quercetin, respectively, showing sustained release of drug by PLA-quercetin nanoparticles [126]. A study by Pool *et al.* demonstrated the presence of hydrogen bonds between quercetin molecules and PLGA [127]. They also revealed that increased drug release occurs in acidic pH, suggesting augmented expulsion of drug in tumor environment. Quercetin-gold loaded PLGA nanoparticles were found to display anti-proliferative activity on HepG2 hepatocarcinoma cells by interacting with cellular DNA and reducing deactylation of histone proteins, arresting the cell growth in the sub-G stage [128]. Orally delivered quercetin, co-encapsulated with tamoxifen, in PLGA polymeric nanoparticles significantly enhanced its bioavailability and suppressed breast cancer growth by inducing apoptosis [129]. In addition, there was no observed hepatotoxicity or oxidative stress compared to free drugs. In another study, quercetin loaded PEGylated PLGA nanoparticles, labeled with folic acid, were intravenously treated to human ovarian adenocarcinoma cells (IGROV-1) and human epithelial cells (HeLa) xenograft models. Researchers observed enhanced uptake of folic acid conjugated nanoparticles by selective tumor cells and suggested their future application in targeted drug delivery [130]. Besides, oral and intravenous delivery of quercetin loaded PLGA nanoparticles, peri-tumoral mode of injection was also reported in4T1 breast cancer xenograft mice model. The study demonstrated effective suppression of tumor growth by quercetin loaded PLGA nanoparticles, compared to control [131]. In addition to chemical synthesis of quercetin loaded PLA nanoparticles, greener approach for PLA nanoparticles synthesis for slow and sustained release of quercetin has also been explored [132]. Despite the fact that PLGA and PLA nanoparticles have shown appreciable advancements in drug delivery, there are noteworthy challenges which need to be addressed. Organic solvents used for the synthesis of PLGA/PLA nanoparticles are always challenging in respect to their complete removal, toxicity and emulsification method. Preparation of PLGA/PLA nanoparticles often requires bulk amount of aqueous phase for initial emulsification of polymeric dispersed phase creating a limitation for large scale production. Additionally, it may also cause variations in size range due to uneven emulsion droplet and PLGA/PLA precipitation. Furthermore, poor quercetin loading is also an issue to be solved. Therefore, there is a need for developing appropriate and convenient technologies to overcome the above mentioned challenges and to produce desired quercetin delivery system. In prospect, these nanoparticles can also be a promising therapeutic candidate for anticancer treatment.

### 5.3. Chitosan Nanoparticles

Chitosan, a natural polycationic polysaccharide, is an *N*-deacetylated derivative of chitin consisting of linear repeating units of 2-acetamido-2-deoxy-d-glucose and β-(1-4)-2-amino-2-deoxy-d-glucose [133]. Owing to the presence of surface amine groups and the modulatory effects on cellular F-actin, tight junction protein ZO-1 and protein kinase C, chitosan is rapidly internalized by cells and is considered an absorption enhancer [134,135,136]. Therefore, it is an attractive candidate for biomedical applications including tissue engineering, drug delivery, wound dressings and antimicrobial activity. As previously reported, chitosan possess anti-inflammatory and antioxidant properties [137,138]. In addition, it also serves as a biocompatible and biodegradable polymer [139]. Being polycationic, chitosan can interact with negatively charged molecules and can form a core shell nanostructure that serves as an efficient drug delivery system [140]. As a drug carrier, chitosan has been reported to be capable of delivering drugs to various organs such as kidney, liver, lungs and colon [141]. A pH sensitive, chitosan-coated nanoparticle system to enhance the oral uptake of drugs via chitosan mediated tight junction opening has also been developed [142]. Moreover, a complex delivery system made up of chitosan and its derivative (*O*-carboxymethylchitosan) has also been synthesized to enhance drug absorption through small intestine involving clathrin-mediated endocytosis [143]. David *et al.*, reported the dose dependent anticancer activity of quercetin-loaded chitosan nanoparticles with and without 5-flourouracil against pancreatic cancer and gave a new insight for the application of quercetin-loaded chitosan nanoparticles for cancer treatment [144]. Interestingly, they also observed low toxicity of dual drug-loaded chitosan nanoparticles against normal L292 cells (murine aneuploidy fibrosarcoma cell line). Their cell internalization study showed the accumulation of dual drug-loaded chitosan nanoparticles in the interior of the cells within 4 h of treatment. Besides anti-proliferative activity, quercetin can also enhance the oral bioavailability of commercially available anticancer drugs such as paclitaxel by inhibiting MDR family members (P-gp, MRP1 and BCRP) and CYP3A subfamily of P-450 cytochrome which can metabolize paclitaxel [145]. Therefore, Wang *et al.*, designed chitosan conjugated quercetin nanoparticles loaded with paclitaxel to enhance oral bioavailability and water solubility via circumventing P-gp efflux pumps [146]. While great advancements have been attained in the development of chitosan-based quercetin delivery system, some issues still need to be fixed before its clinical translation. Majorly, the biocompatibility of modified chitosan derivatives, used as quercetin carriers, should be explored in more detail. Additionally, the absorption and bioavailability of quercetin carried by chitosan or its derivatives can be further improved.

### 5.4. Liposomes

Liposomes consist of amphiphilic lipid molecules which can form a bilayer membrane spherical vesicle, well known to carry therapeutic and diagnostic cargos to their site of action [147,148]. Liposomes extravagate through the pores present in the capillary endothelium, found especially in tumor cells, and accumulate at the tumor sites [149]. Because of the presence of a lipid bilayer and an aqueous core, liposomes are capable of carrying and transporting both hydrophobic and hydrophilic therapeutic agents [150]. The physicochemical properties of liposomes such as size, surface charge, composition and targeting ligand can be modulated according to the drug, site of action and the disease, making it feasible for controlled and specific delivery system [151]. With the help of targeted drug delivery, liposomes have been employed to deliver low doses of drugs with reduced toxicity and side effects. Wang *et al.*, reported enhanced cytotoxic effect of quercetin-loaded liposomes in C6 glioma cells. Additionally, they concluded the involvement of necrosis, rather than apoptosis, during tumor cell death [152]. Increased bioavailability, cellular uptake and aqueous solubility of quercetin loaded nanoliposomes were also reported using MCF-7 human breast cancer cells. Their data suggested the use of quercetin-loaded liposomes as an efficient antioxidant [153]. Recently, Soluplus micelles, an amphiphilic polyvinyl caprolactam-polyvinylacetate-polyethylene glycol graft copolymer, have been proposed to encapsulate poorly water soluble drugs for efficient bioavailability [154]. The study reported enhanced oral bioavailability of quercetin in beagle dogs after oral administration of quercetin-loaded polymeric micelles (relatively 286% more oral bioavailability compared to free quercetin). However, liposomes have certain limitations as a drug carrier including low drug loading, less stability, high cost and active targeting. This necessitates further research in designing innovative routes and methods for developing potent quercetin liposomal formulations as an anticancer agent.

### 5.5. Other Nanoparticles

To improve the efficacy of orally administered quercetin, Tan *et al.*, employed polyethylene glycol (PEG)-derivatized phosphatidyl ethanolamine (PE) as a block copolymer for the synthesis of quercetin nanomicelles [155]. They further evaluated the interaction of nanomicelles with Caco-2 cells and studied their anti-proliferative effect on A549 lung cancer cells and a mice xenograft model. Quercetin nanomicellar solution exhibited suppressive tumor growth with a margin of 1.5 fold higher than free quercetin, displaying no observed toxicity in relevance to weight loss [155]. A novel approach to enhance the solubility of quercetin by fabricating quercetin nanoribbons by atmospheric pressure physical vapor deposition (PVD) approach has also been successfully attempted. The increased growth inhibitory effect of quercetin nanoribbons on 4T1 breast cancer cells also demonstrated their improved aqueous solubility and drug release profile [156]. Nevertheless, further studies are still required to understand the underlying mechanism of anticancer activity of these quercetin nanoformulations.

## 6. Future Perspectives and Limitations

The pharmacological effect of quercetin loaded/conjugated nanoparticles majorly depends on the drug carriers used and the physico-chemical properties of the nanoparticulate system. These characteristics can increase the stability of quercetin, its bioavailability and target specificity. Hence, an overview of the physicochemical properties and biological efficacy of some of the discussed nanoparticles are detailed in Table 2. Despite numerous *in vitro* and *in vivo* anti-cancer studies of quercetin nanoformulations, there are still some limitations for their clinical translation such as cost, safety and side effects [157]. The *in vivo* anticancer potential of quercetin nanoparticles have been evaluated on various kinds of cancer models among which oral administration is the most preferred route for quercetin nanoformulation administration [158]. Although various quercetin nanoparticles capable of enhancing quercetin bioavailability in the body have been synthesized, the need for more stable and target specific nanoparticles is still a challenging issue [159]. 

Also, a reduction in the side effects and toxicity of quercetin nanoparticles should be considered before they are applied for clinical purposes [160]. This can be achieved by the addition of cancer cell specific targeting moieties on the nanoparticles. It will not only enhance target specific delivery of nanoformulations but will also reduce their interaction with the normal cells, preventing any side effects of anticancerous drugs. Additionally, more controlled synthesis of nanoformulations will be required to fulfill the safety criteria specified by government authorities [161]. Furthermore, before entering the market as therapeutic agent, cost effectiveness of the nanoformulations will be a major issue and cheaper and affordable drugs are required.

## 7. Conclusions

Multifunctional quercetin, a dietary flavonoid, is a competent antioxidant and can serve as a potent anti-cancer agent. However, its efficiency as a therapeutic drug is low due to its rapid metabolism in the body. Thus, the use of biodegradable and biocompatible carriers as delivery systems can enhance the therapeutic competence of quercetin. Some of the most well-studied and available quercetin delivery systems are based on liposomes, PLGA, PLA, chitosan and silica. Both *in vivo* and *in vitro* studies have highlighted the anti-proliferative effect of quercetin-loaded nanoparticles on various types of cancer cells. However, the use of such nanoparticles at a clinical level still needs more process optimization to enhance their specificity and efficacy for their efficient clinical translation.

**Table 2:** The physico-chemical properties and anti-cancer effects of quercetin based nanoparticle systems.

Delivery System	Cancer Type	Chemicals/Polymer Used	Size (d) nm	PDI	Entrapment Efficiency	*In Vitro*/*In Vivo*	Effective Dose	Effect	Ref.
Silica	JR8 human melanoma cell line	aminopropyl functionalized mesoporous silica nanoparticle	250 ± 50	NA	NA	*In vitro*	60 µM	~50% inhibition of cell proliferation at 72 h	[117]
						*Ex vivo* (Porcine skin)	0.27% *w*/*w* of quercetin in water/oil emulsion system	Higher amount of quercetin was retained in the skin as compared to control at 24 h	
PLGA/PLA	A549 human lung adenocarcinoma epithelial cell line	PLGA (combination treatment of quercetin and etopside)	153.4 ± 4.2 (etopside), 148.6 ± 1.6 (quercetin)	0.058 ± 0.02 (etopside), 0.088 ± 0.03 (quercetin)	63.88% ± 1.5% (etopside), 41.36% ± 3.4% (quercetin)	*In vitro*	50 µM	Enhanced cytotoxic effect compared to free drugs combination at 72 h	[125]
	MDA-MB231 human breast cancer cell line	PLA	46 ± 6	NA	62% ± 3%	*In vitro*	100 µg/mL	~40% decrease in cell viability in 5 days	[126]
	DMBA induced Breast cancer	PLGA (coencapsulated quercetin and tamoxifen)	185.3 ± 1.20	0.184 ± 0.004	67.16% ± 1.24% (tamoxifen), 68.60% ± 1.58% (quercetin)	*In vitro*	10 µg/mL	increase in cell cytotoxicity	[129]
						*In vivo* (female SD rats)	45 mg/kg (Oral, one time per week for 3 weeks	Tumor was reduced to ~32.36% after 30 days	
	HeLa cervical-tumor-derived cell line or IGROV-1 human ovarian carcinoma cell line	PEG-PLGA and Folic acid as targeting ligand	155.0 ± 1.2	<0.2	97.8 ± 0.14	*In vitro*	10 µM	~56.63% reduction in cell viability of HeLa	[130]
						*In vivo* (female athymic nude and SHrN mice xenograft model)	250 μL of 50 mg polymer/mL (single intravenous injection)	Folic acid enhances selective uptake of nanoparticles by folate receptor enriched cancer cells	
	MDA-MB-231 human breast cancer cell line and 4T1 murine mammary cancer cell line	MPEG-PLA	155.3 ± 3.2	0.2 ± 0.05	NA	*In vitro*	13.5 µg/mL	~38% lower cell viability compared to control	[131]
						*In vivo* (female BALB/c mouse xenograft model)	0.5 mg/kg (peritumoral injection, every third day till day 19)	Reduced tumor size as compared to control	
Chitosan	MiaPaCa2, Pancreatic cancer cell line	Chitosan	300	NA	91%	*In vitro*	10 µM to 100µM	Dose dependent cell inhibition	[144]
	MiaPaCa2, Pancreatic cancer cell line	Chitosan (quercetin and 5-flourouracil dual drug loading)	400	NA	95% (quercetin), 75% (flourouracil)	*In vitro*	39.7 µM (quercetin) and 75 µM (flourouracil)	~70% decrease in cell viability	[144]
	HepG2 human liver cancer cell line	Chirosan-quercetin conjugate loaded with paclitaxel	185.8 ± 4.6	0.134 ± 0.056	85.63% ± 1.26%	*In vitro*	0.01–100 µg/mL	Dose dependent cytotoxic effect with IC_50_ 0.11 µg/mL	[146]
						*In vivo* (male ICR xenograft models)	20 mg/kg (single oral dose)	~71.22% reduction in tumor size	
Liposomes	C6 glioma cell line	glyceryl behenate, soy lecithin, and cholesterol	116.7	NA	NA	*In vitro*	0–400 μM	Induced necrotic cell death	[152]
	MCF-7 human breast cancer cell line	Phosphotidyl choline	100.974 ± 0.3	NA	40.7% ± 3.1%	*In vitro*	50 mM/mL	~83% inhibition in cell proliferation at 48 h	[153]
	MCF-7 and MDA-MB-231 human breast cancer cell line	Soy lecithin, glyceryl tridecanoate, glyceryl tripalmitate, vitamin E acetate, Kolliphor HS15	32	0.059	95%	*In vitro*	50 µM	~13.7% reduction in viability of MCF-7 cells and ~13.4% reduction in viability of MDA-MB-231 cells at 48 h	[162]
Nanomicelles	A549 human lung cancer cell line	DSPE-PEG_2000_ Nanomicelles	15.4–18.5	<0.250	≥88.9%	*In vitro*	100 µM	Decreased cell viability at 72 h	[155]
						*In vivo* (female Rag-2M mice xenograft Model)	30 mg/kg (three times per week for 3 weeks, perorally)	~1.5 fold higher tumor growth inhibition than free quercetin control group	
Nanoribbon	4T1 murine mammary cancer cell line	Nanoribbon fabricated by atmospheric pressure PVD	100–200	NA	NA	*In vitro*	NA	~57% reduction in cell viability	[156]

NA: not available; PDI: poly dispersity index; d: diameter.
